# I-AbACUS: a Reliable Software Tool for the Semi-Automatic Analysis of Invasion and Migration Transwell Assays

**DOI:** 10.1038/s41598-018-22091-5

**Published:** 2018-02-28

**Authors:** Marilisa Cortesi, Estelle Llamosas, Claire E. Henry, Raani-Yogeeta A. Kumaran, Benedict Ng, Janet Youkhana, Caroline E. Ford

**Affiliations:** 1 Laboratory of Cellular and Molecular Engineering “S. Cavalcanti”, Department of Electrical, Electronic and Information Engineering “G. Marconi” (DEI), University of Bologna, Cesena, Italy; 2 Gynaecological Cancer Research Group, Lowy Cancer Research Centre and School of Women’s and Children’s Health, Faculty of Medicine, University of New South Wales, Sydney, Australia; 3 Adult Cancer Program, Lowy Cancer Research Center, Prince of Wales Clinical School, University of New South Wales, Sydney, Australia

## Abstract

The quantification of invasion and migration is an important aspect of cancer research, used both in the study of the molecular processes involved in this collection of diseases and the evaluation of the efficacy of new potential treatments. The transwell assay, while being one of the most widely used techniques for the evaluation of these characteristics, shows a high dependence on the operator’s ability to correctly identify the cells and a low protocol standardization. Here we present I-AbACUS, a software tool specifically designed to aid the analysis of transwell assays that automatically and specifically recognizes cells in images of stained membranes and provides the user with a suggested cell count. A complete description of this instrument, together with its validation against the standard analysis technique for this assay is presented. Furthermore, we show that I-AbACUS is versatile and able to elaborate images containing cells with different morphologies and that the obtained results are less dependent on the operator and their experience. We anticipate that this instrument, freely available (Gnu Public Licence GPL v2) at www.marilisacortesi.com as a standalone application, could significantly improve the quantification of invasion and migration of cancer cells.

## Introduction

Migration and invasion are hallmarks of cancer as they allow cells to abandon their site of origin and spread to other tissues of the body, leading to the development of metastasis^1,2^. The study and quantification of these processes are of paramount importance in cancer research, in both identifying potential therapeutic targets and understanding the molecular processes that influence metastasis^3,4^.

In this work the focus is on the *in-vitro* study of migration and invasion and specifically on the transwell assay, one of the most common experiments used to quantify these processes^3–6^, with 4198 papers in the last five years associated with the keywords “transwell assay” or “Boyden chamber” on Pubmed (database accessed in January 2017). This common assay consists of seeding a defined number of cells on a cell permeable membrane, that when studying invasion, is coated with a layer of Matrigel to simulate the extracellular matrix. The invasiveness of the population is quantified as the number of cells that are able to cross the membrane within a specific time frame.

While easy to implement and relatively straightforward to interpret, this assay has a number of drawbacks, the most important being that it is highly dependent on the individual operator and their level of experience. This is due to the fact that the cells that have invaded through the membrane are manually counted, either from images acquired with an optical microscope, using image processing software that implement object counting functionalities^3,6^, or directly from the instrument, with the help of a mechanical cell counter^2,4,5,7^. As a consequence results can vary significantly with the operator’s ability to distinguish the cells from debris and pores, a task that can be quite complex especially as the number of cells in each field increases or if they exhibit a range of different phenotypes.

Another important aspect is the lack of standardization of the assay. The experimental protocol can vary significantly in the number of fields considered for each membrane, in the magnification of the objective used to visualize them and in the optimal cell density, that is the minimum number of cells/field that would allow to correctly capture the behavior of population. The combination of these factors prevents the direct comparison between results obtained by different research laboratories and limits the accuracy and reproducibility of the results. A computational tool able to automatically identify the cells could address these limitations and improve the analysis of transwell invasion/migration assays. While a number of cell segmentation algorithms have been developed, they tend to be optimized for specific applications and thus potentially unreliable in different contexts. For example, in refs^8,9^ computational tools for the analysis of immunohistochemistry images are presented. The high level of organization of the tissue samples and the clustering of the cells, led to the development of algorithms specific for tessellated images and partially superimposed objects, that might not be effective when the regions of interest are completely separate or when the background is not uniform, as in the present case, due to the presence of pores. Other systems rely on characteristics specific of the target images, like signal patterns generated by the cellular membranes^10^, or the presence of a single cell^11^. Consequently, current tools are highly effective but only within the application they’ve been developed for. A more general approach is presented in^12^ where the user is required to annotate the images and verify the algorithm’s result. While being more versatile, this technique requires an extensive human intervention that might make it excessively time consuming for the analysis of transwell assays, where a large number of images must generally be segmented.

To address these limitations and improve the *in-vitro* quantification of migration and invasion, we have developed I- AbACUS (Invasion Assay Assisted Cell coUnting Software) a custom-made software that allows users to standardize the counting process, applying the same procedure to every image. I-AbACUS is coded in Matlab R2016a (The Mathworks, Inc., Natick, Massachusetts, USA) and is freely available both as source code and standalone application. It integrates an intuitive graphical user interface (GUI), that enhances its usability and the segmentation parameters can be easily changed, to extend its applicability to cells with different sizes and morphologies. Finally, a learning algorithm has been implemented within I-AbACUS to improve the recognition of specific kinds of cells.

Here, we compare I-AbACUS and the standard approach for the analysis of transwell assays, and demonstrate the key advantage of I-AbACUS in overcoming the diversity of operator, making the results obtained with I-AbACUS reliable and robust.

## Results

### Equivalence between I-AbACUS and the manual count

**Figure 1:**
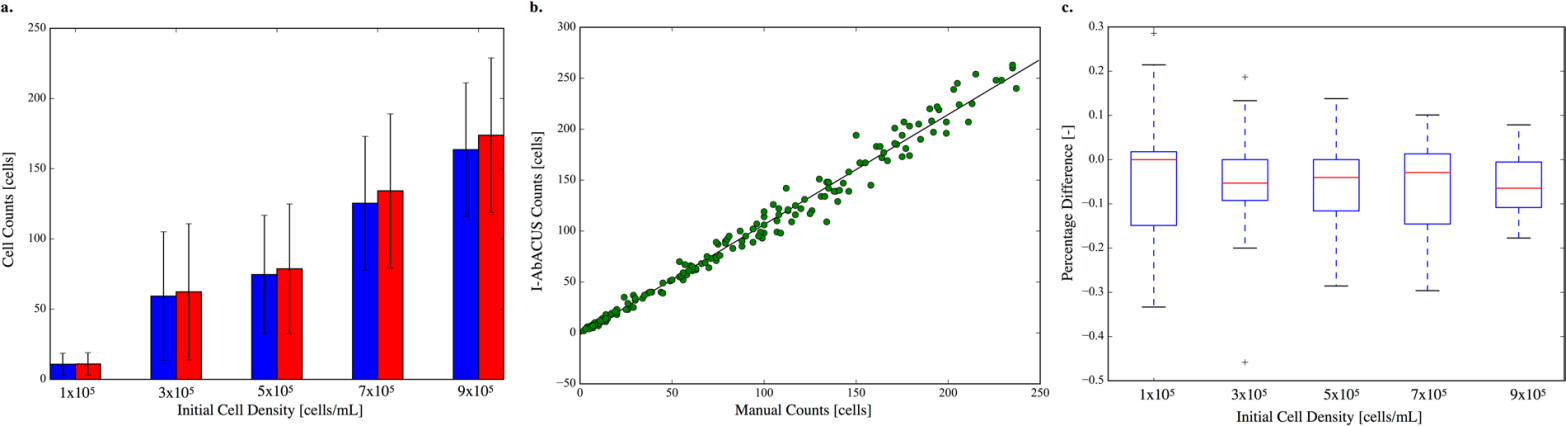
Comparison between I-AbACUS and the standard technique for the analysis of transwell assay, the manual count. A dataset of 180 images of OVCAR4 cells was elaborated with both methods and the results were compared. In (**a**) the relation between average cell counts and initial cell density is shown. The manual counts are represented in blue, while the ones obtained with I-AbACUS are in red and the errorbars represent the standard deviation. Panel **(b**) considers the agreement between the two techniques on single images. The correlation between the two measures is excellent (R^2^ = 0.992) and stable over the tested conditions. In (**c**) the percentage difference between the I-AbACUS counts and the manual ones is reported as a function of the initial cell density. The result dependence on the number of cells is low, since this parameter remains approximately constant and the average error is below 10%.

The first step in the validation of a new tool or instrument is the comparison of its results with that obtained with the gold standard technique. Thus, the same images (n = 180) were analyzed both with I-AbACUS and with the traditional method, by the same expert operator. For this test five different initial concentrations of OVCAR4 ovarian cancer epithelial cells were seeded in the Matrigel coated transwells and invasion was determined after 48 hours. Both total cell counts and counting times were recored. In the traditional analysis (in blue in Fig. 1a) the object counting functionality of ImageJ was used, while with I-AbACUS (in red in Fig. 1a) the images were elaborated following the standard workflow described in the Methods section.

The cell counts showed very good agreement considering both the average cell count for each condition (Fig. 1a) and single images (Fig. 1b), where the Pearson’s correlation coefficient between the manual and the I-AbACUS counts was 0.992. The error bars in Fig. 1, that represent the standard deviation of the cell counts for the corresponding condition, have comparable amplitude for the two methods. The percentage difference, computed considering the manual count as the theoretical value, didn’t change considerably between the tested conditions, thus demonstrating the equivalence of the two methods on a wide range of cellular densities (Fig. 1c).

**Figure 2:**
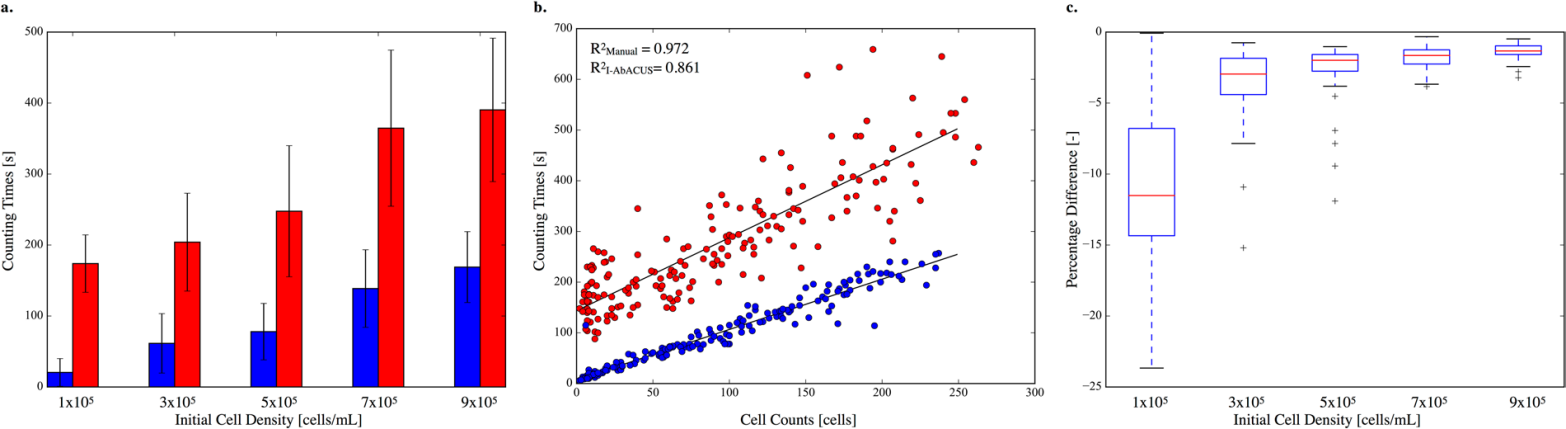
Analysis of the counting times recorded while obtaining the results of Fig. 1. The time required for the analysis of each image with both techniques was measured and in (**a**) its average value and standard deviation is shown, as a function of the corresponding initial cell density. In blue are represented the results obtained with the manual counting while red identifies the I-AbACUS’s ones. In (**b**) the relation between counting times and cell counts is reported. While both methods show a correlation between these two variables, the time required for the analysis with I-AbACUS has a weaker dependence on cell number (red dots, R^2^ = 0.861). The manual analysis (blue dots, R^2^ = 0.972), on the other hand, is characterized by an almost constant counting rate (number of cells counted per second). In (**c**) the percentage difference between the I-AbACUS counting times and the corresponding manual ones are reported. The increase in initial cell density is associated to a marked decrease of this parameter (almost 10 fold change between 1 × 10^5^ and 9 × 10^5^ cells/mL). This demonstrates how the use of I-AbACUS is particularly convenient at high cell density, when the manual count becomes less reliable and more time consuming.

Average counting time was reported as a function of the initial cell density. The analysis with I-AbACUS (in red in Fig. 2a and b) took consistently longer than the manual one (in blue in Fig. 2a), partly due to the fact that the time required by the program to analyze the images was included in the measure, thus introducing a non-negligible offset (Fig. 2a). This is also represented in Fig. 2b where the dependence of the counting time on the number of cells in the image is considered. The traditional analysis showed a very good correlation between these two variables (R^2^ = 0.972), revealing how the number of cells counted per time unit was almost independent of the total number of cells in the image. On the other hand, the I-AbACUS counting times showed a lower dependence on the image density (R^2^ = 0.861) and an offset of about 120 s. The exclusion of the elaboration time, however, would have reduced the accuracy and the correctness of the comparison, since the image’s elaboration is an integral part of the I-AbACUS workflow.

Figure 2c reports the percentage difference between the counting times recorded with the two methods. It markedly decreased with the initial cell density (almost 10-fold comparing 1 × 10^5^ and 9 × 10^5^ cells/mL), showing how the inconvenience of a non-negligible elaboration time can be compensated, exploiting the lower dependence of the I-AbACUS counting times on the cell density. Since the percentage difference on the cell count wasn’t influenced by the initial density (Fig. 1c), considering images with a higher number of cells would increase the efficiency of the analysis, reducing the incidence of the elaboration phase on the total counting time, while increasing the robustness of the results, due to the increase in population size.

### Comparison between cell identification methods

**Figure 3:**
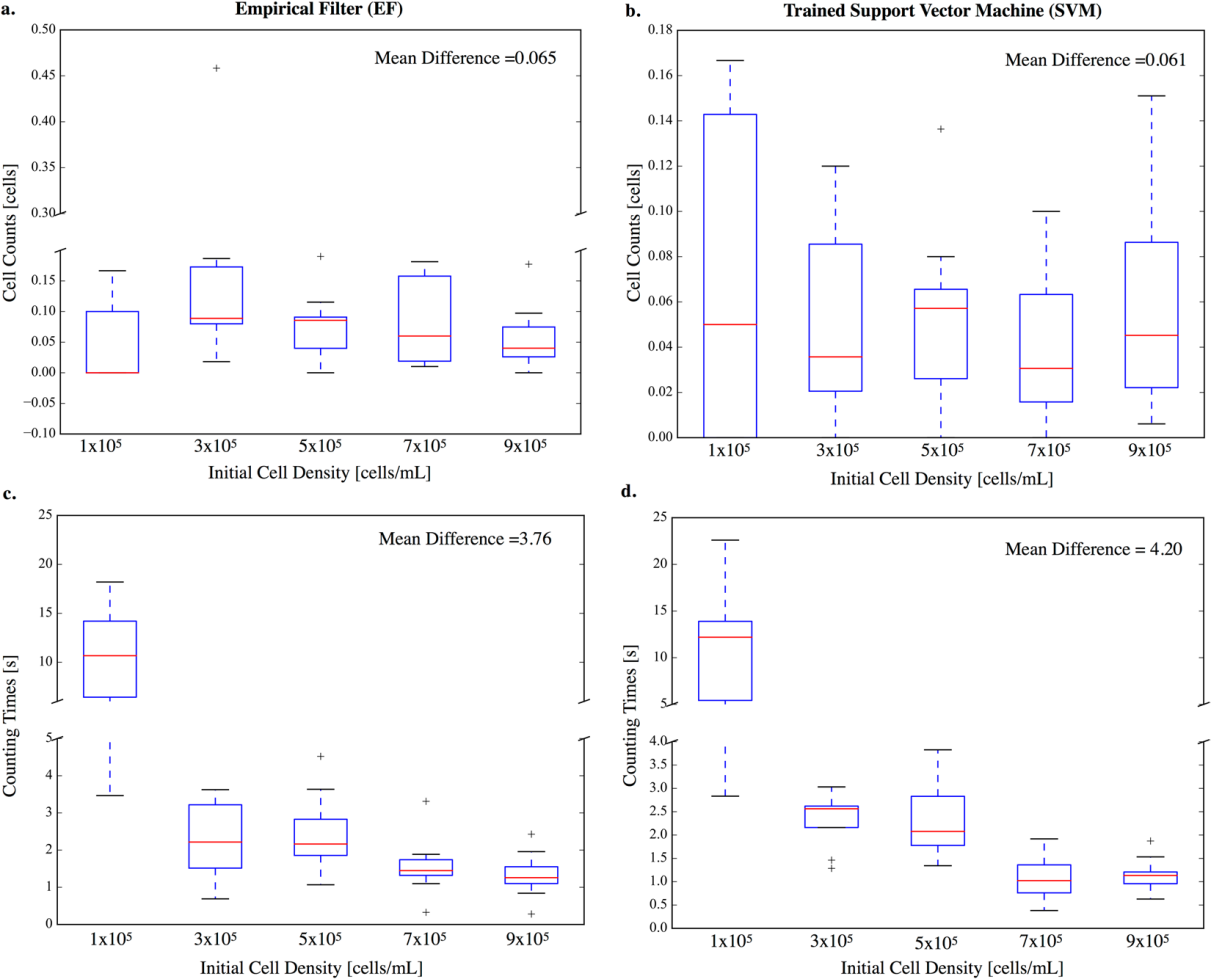
Comparison between the two cell identification methods implemented in I-AbACUS. The dataset of OVCAR4 cells used in the previous analysis was used to compare the two cell selection methods. Panel **(a)** reports the percentage difference between the manual cell counts and those obtained with the EF of I-AbACUS. It remains approximately constant throughout the tested conditions and its average value is 0.065. The same analysis is shown in (**b**) using the trained SVM as cell selection method. While the average percentage difference between the two methods does not markedly change, with respect to the data reported in panel (**a**), the mean difference distribution is narrower and the number of outliers is reduced. In (**c**) and (**d**) the counting times recorded while generating the data shown, respectively, in (**a** and **b**) is reported. The use of the SVM is associated to a slight increase in analysis time (mean percentage difference 4.20), with respect to the EF method (mean percentage difference 3.76).

The ability to distinguish cells from pores and debris is one of the most important functions of I-AbACUS. It was implemented using two alternative approaches, an Empirical Filter (EF) that can be generally applied to different kinds of cells and a trained Support Vector Machine (SVM) that can be used to improve the classification of a specific type of cells. Their performances were compared using the same dataset used to demonstrate the equivalence with the manual count. The same operator again analyzed the images using a SVM trained on a completely independent set of images, composed of 160 images, 48 of which were acquired from membranes on which A2780 ovarian cancer cells were seeded, while the others were obtained from experiments involving OVCAR4 cells. Figure 3 reports the results of this comparison as the percentage difference between the results obtained with I-AbACUS (EF or SVM) and the manual count.

The use of the SVM didn’t significantly affect the counting time (Fig. 3c and d) or the average cell count (Fig. 3a and b), however it increased the reliability of the count reducing the number of outliers and their distance from the rest of the population.

### Inter-operator variability

One of the most critical aspects of the traditional analysis of transwell assays is the high inter-operator variability, meaning that the results vary significantly with the operator’s ability to correctly identify the cells.

To test if the use of I-AbACUS could address this limitation we asked five other operators to count two sets of 22 and 23 images respectively, extracted from the one used for the previous comparisons and covering all the tested initial cell densities. One operator for every level of experience was assigned to the first dataset while the other naive and medium experienced operators counted the second group of images. They were asked to count the cells in the provided images both with I-AbACUS, using the EF and with the traditional approach. Furthermore, to eliminate any possible confounding factor all the users were given the same set of instructions on how to count cells, both manually and with I-AbACUS.

**Figure 4:**
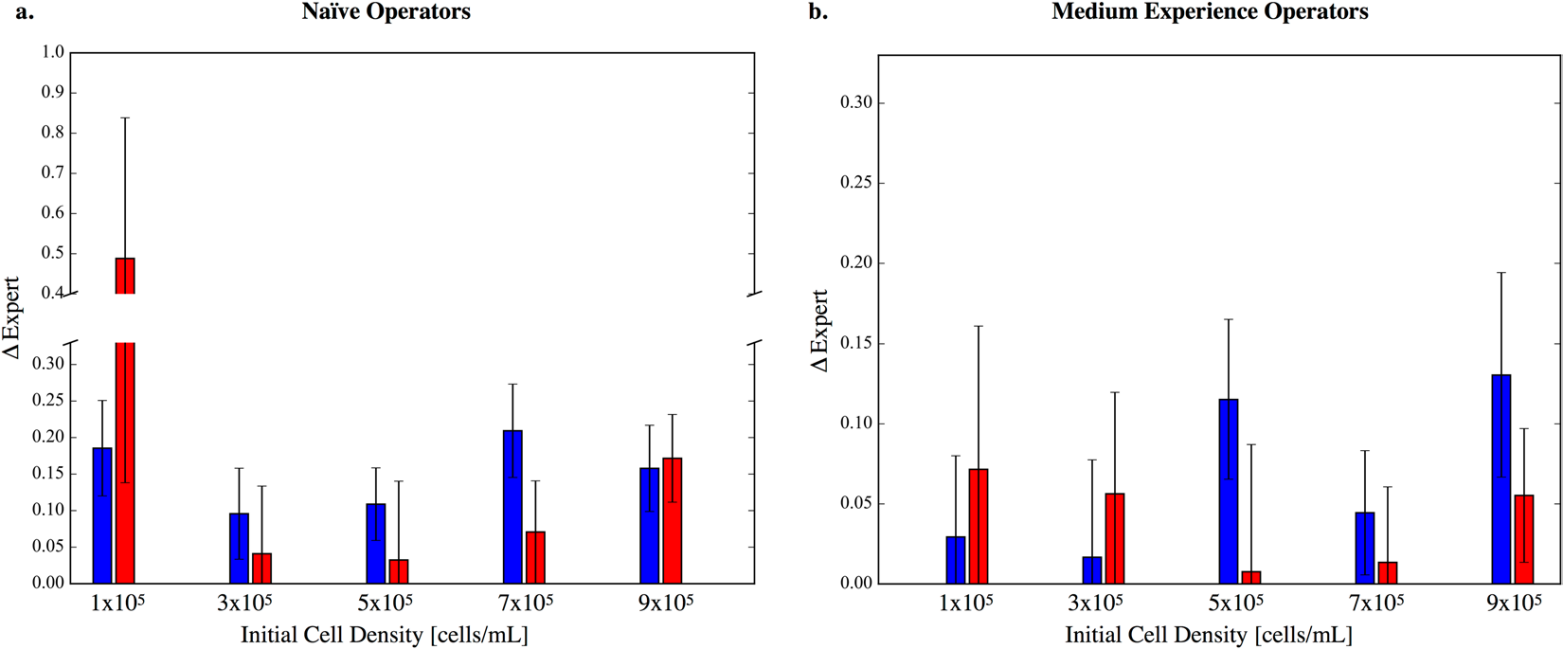
Analysis of the inter-operator variability and effect of the user experience on the results. A subset of about 20 images, extracted from the dataset used in the previous analyses, was elaborated by 5 additional people with different levels of experience with transwell assays. In (**a**) the difference between the results obtained by the nalve operators and the expert are shown. Blue identifies the data obtained with the traditional analysis (manual count) while in red are reported the I-AbACUS’s results. These were determined to be statistically different (Welch’s t-test, p < 0.05). (**b**) Same graph as in **(a)** but with the data obtained by the medium experience operators. In the case the results obtained with I-AbACUS were not statistically different from the manual ones.

Figure 4 shows the results of this test as difference between the average cell counts obtained by the naive and medium operators and that of the expert (Fig. 4a and b respectively). All the data were normalized with respect to the values obtained by the I-AbACUS expert operator, that produced the results of the previous comparisons.

The use of I-AbACUS (red bar) reduced, in most cases, the difference between the counts obtained by the expert operator and those of less skilled users. This difference was statistically significant (p < 0.05) when the data produced by the naive operators were considered, showing how being provided with a suggested result decreases the inter-operator variability and the dependence of the results on the user’s experience. This is particularly evident when looking at an initial cell density of 5 × 10^5^ and 7 × 10^5^ cells/mL, which can be considered to be the target cellular density for transwell invasion assays with OVCAR4 cells. The error of the experienced operator will be minimized, since this is the type of images on which she has more training, while the other operators will find them challenging due to the increased number of cells.

### Intra-operator variability

Intra-operator variability refers to the difference in the number of cell counted by the same operator, when repeating the analysis of an image. Low intra-operator variability is a desirable characteristic as it is associated to the assay’s repeatability and the robustness of the results. To evaluate if the use of I-AbACUS could improve this aspect, one operator for each level of experience counted again a subset of images (9) from the ones used in the inter-operator variability test. In this case only 3 initial cell densities were considered (1 × 10^5^, 5 × 10^5^, 9 × 10^5^ cells/mL) and the operators were asked to execute the analysis as in the previous test.

**Figure 5:**
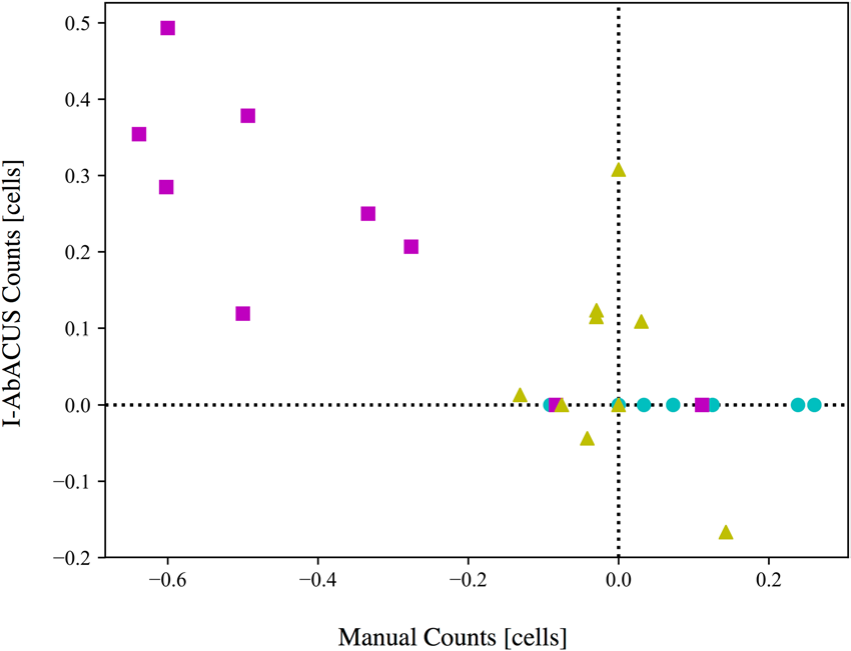
Study of the intra-operator variability. Nine of the images used in the analysis of the inter-operator variability were elaborated again by three operators and the percentage difference between the counts obtained on the same image, with either method. The expert operator was shown to be the most consistent, as her results (yellow triangles) are clustered around zero,. The nailve operator, identified with the cyan circles, refrained from modifying the cell count proposed by I-AbACUS, as a consequence the percentage difference on the y axis is always 0. The medium experience operator (magenta squares) shows the higher variability, that however is reduced when using I-AbACUS.

Figure 5 reports the results of this test as a scatter plot in which the percentage difference between the two counts obtained for the same images are reported. The use of I-AbACUS significantly reduced the intra-operator variability, measured as the median percentage difference between the two counts (Table 1).

**Table 1:** Median percentage difference between the two counts obtained by the same operator on the same images. The use of I-AbACUS reduces the intra-operator variability improving the assay’s repeatability.

	Manual Count	I-AbACUS Count	Marker
Naive operator	11.1%	0%	cyan circle
Medium operator	−49.3%	25%	magenta square
Expert operator	−2.9%	1.3%	yellow triangle

In this test the naive operator did not modify the cell count proposed by I-AbACUS thus leading to exactly the same result for every image. Furthermore, the intra-operator variability seems to have a lower dependence on the level of experience of the operator, since the results of the medium experience operator are less robust than the ones of the naive user.

### Versatility

To test the ability of I-AbACUS to analyze images containing cells other than OVCAR4, a dataset of 16 images was acquired using A2780 cells (initial cell density 5 × 10^5^ cells/mL) that display a more diverse phenotype and a more mesenchymal-like morphology (Fig. S1).

**Figure 6:**
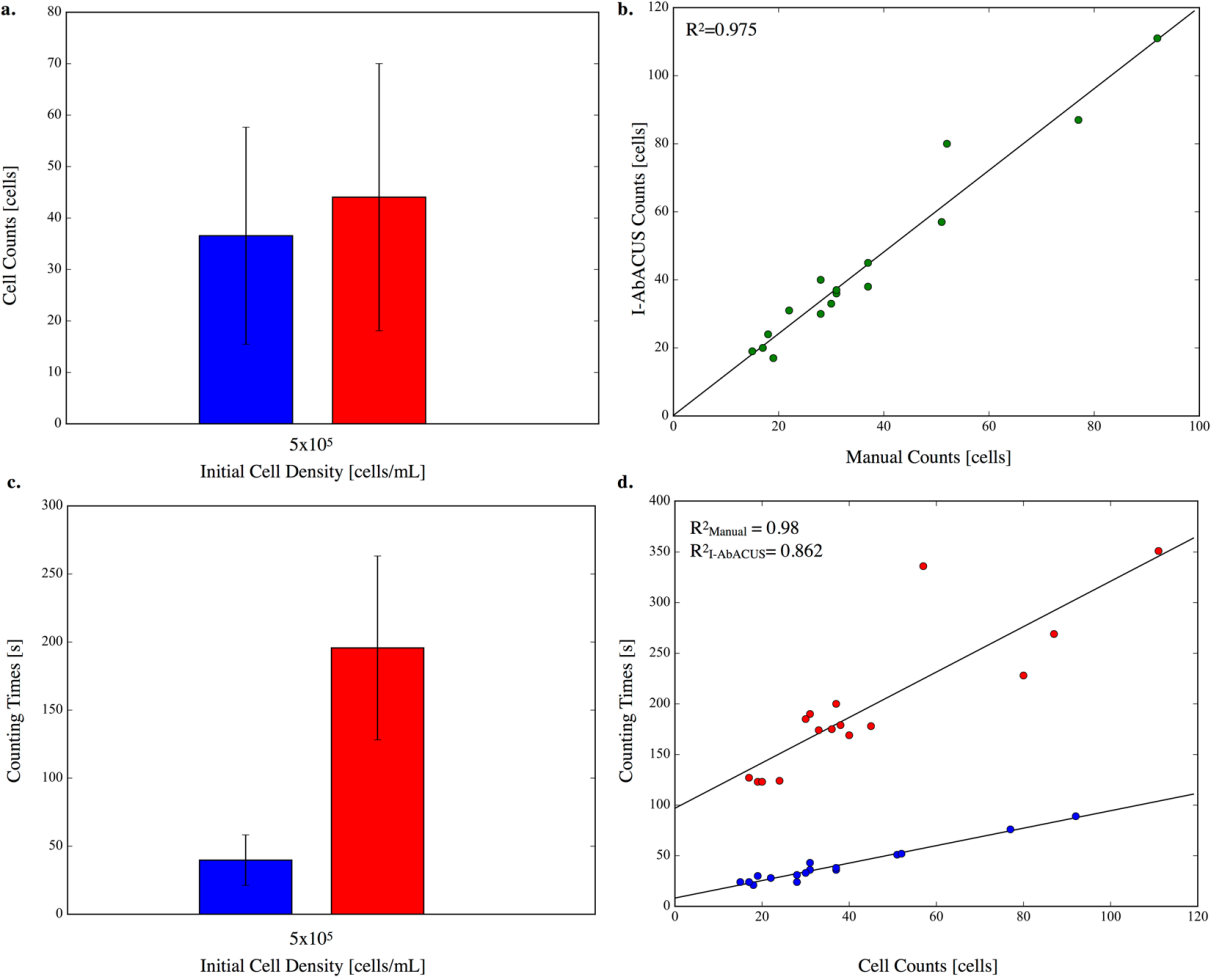
Evaluation of I-AbACUS’s versatility and its ability of recognizing cells with different morphologies. In this case a dataset of 16 images of A2780 cells was used and a single cell density (5 × 10^5^ cells/ml) was considered. In (**a**) the average cell count obtained with I-AbACUS (in red) and the standard technique (in blue) are compared through a bar chart reporting mean and standard deviation. This analysis is complemented by the evaluation, in (**b**) of the concordance obtained on single images. The results of the two methods are highly correlated (R^2^ = 0.975) and well approximated by the linear regression obtained applying the least square method. In (**c**) the average counting time recorded while obtaining the results presented in (**a**) is shown, together with the corresponding standard deviation. The scatter plot in (**d**) analyses the relation between counting times and cell counts. The data obtained with the manual counting, reported in blue, have a higher correlation (R^2^ = 0.980) than the I-AbACUS ones (R^2^ = 0.862, red circles). These results closely mirror the ones in Figures 1 and 2, albeit with a higher variability probably caused by the smaller dataset size.

The same experienced user counted all the images, both with the traditional approach (in blue in Fig. 6a and c) and with I-AbACUS (in red in Fig. 6a and c), using the EF as classification method.

Figure 6 reports the results of this analysis that closely mirror the ones in Figs 1 and 2, the average cell counts were equivalent (Fig. 6a) percentage difference ~20%) and the correlation between the results obtained on the single images was very high, R^2^ = 0.975 (Fig. 6b).

The counting time was still significantly lower when the images are analyzed manually but again its dependence on the image density was lower with I-AbACUS (Fig. 6c, ${{\rm{R}}}_{I-\mathrm{AbACUS}}^{2}=0.862$ and ${{\rm{R}}}_{{\rm{Manual}}}^{2}=0.980$) suggesting the possibility of incrementing the initial cell density to increase the efficiency of the test and the robustness of the results, as shown for the OVCAR4 data. Being able to elaborate, using the same parameters, images featuring cells with different morphologies, greatly expands the I-AbACUS applicability, not only to different cell lines but also to the analysis of invasion assay that use treatments that modify the morphology of the cells.

## Discussion

Here we have presented I-AbACUS, a computational tool designed to aid the analysis of transwell invasion/migration assays through the automatic segmentation of the cells and the suggestion of a cell count.

We have demonstrated the equivalence between the I-AbACUS results and those obtained with standard techniques on a wide dynamic range (Fig. 1) and we have shown that I-AbACUS is able to address the major disadvantages of manual counting. Specifically, it reduces the inter-operator variability and the dependence of the results on the operator’s experience (Fig. 4). Furthermore, it improves the robustness and the reproducibility of the assay by decreasing the intra-operator variability (Fig. 5).

I-AbACUS has been designed to be versatile and adapt to the analysis of images of cells with different morphologies (Fig. 6), however the classification of a specific cellular type can be improved by exploiting the learning algorithm integrated within the program (Fig. 3).

Besides improving the analysis of transwell assays, increasing the repeatability and robustness of their results, I-AbACUS will be developed further to address limitations of the current analysis and generally improve the quantification of the invasiveness of cancer cells.

One of the features that will be included in I-AbACUS is the de-identification of the images, to avoid biases connected to the expected results. After defining the experiment setup, all the images will be loaded and randomly presented to the user, that will not be able to identify the corresponding experimental condition.

Another future improvement of I-AbACUS is the development of a framework that could help researchers in the troubleshooting phase of the invasion/migration transwell assay. Specifically, the determination of the initial cell density is a crucial aspect of the experiment design and it is generally optimized empirically through a trial and error process. A standard calibration protocol will be integrated within I-AbACUS that, through a defined set of experiments, will determine the optimal initial cell density, as the one that would minimize the counting error while leading to the acquisition of enough data to obtain robust results.

Another feature of I-AbACUS that will be developed is a training program, to teach new operators how to analyze images from transwell assays. Users will be provided with a set of images, specifically chosen to highlight the main difficulties of the analysis of these experiments. The comparison between the regions classified as cells by the naive user and those of a trained operator, will allow the program to provide feedback and suggestions on how to improve the trainee’s results.

Finally, to maximize I-AbACUS’s diffusion and application, it is released freely under the Gnu Public License (GPL v2) and can be downloaded at www.marilisacortesi.com.

## Methods

### Cell culture

Two ovarian cancer cell lines, OVCAR4 and A2780, were used in this study. The former was a kind gift of Dr. Michelle Henderson (Children’s Cancer Institute, UNSW, Sydney, Australia) and it has been originally purchased from the American Type Culture Collection (ATCC, Manassas, VA, USA). It was authenticated in September 2016 by the Garvan Institute of Medical Research (Darlinghurst, Sydney NSW) through the analysis of the microsatellite profile. The latter was generously provided by Dr. Elizabeth Roundhill (Children’s Cancer Institute, UNSW, Sydney, Australia) in 2014. They were both cultured in RPMI-1640 media (Thermo Fisher Scientific, Waltham, Massachusetts, USA), supplemented with 10% fetal bovine serum, penicillin/streptomycin and GlutaMAX (Life Technologies, Carlsbad, CA, USA). Cells were grown at 37 °C in 5% CO_2_ and were routinely tested negative for mycoplasma contamination. Examples of each cell line morphology are shown in Fig. S1.

### Transwell invasion assays

In this assay transwell inserts (Corning Life Sciences, Tewksbury, MA, USA) were precoated with 50 μL of Matrigel (Corning Life Sciences, Tewksbury, MA, USA) at a concentration of 1 mg/mL and polymerization allowed to occur at 37 °C in 5% CO_2_ for at least 4 h.

Transwell assays were performed as previously described^3^. Cells were harvested with trypsin and concentration was automatically determined (Countess, Thermo Fisher Scientific, Waltham, Massachusetts, USA), Migration was evaluated at 5 different initial densities for OVCAR4 cells (1 × 10^5^, 3 × 10^5^, 5 × 10^5^, 7 × 10^5^, 9 × 10^5^ cells/ml), and at 5 × 10^5^ cells/ml for A2780 cells.

Cells were seeded in the transwell and after 48 h of incubation were fixed with 100% methanol and stained with crystal violet (0.1%). The membranes were then removed from the transwell and mounted on a microscope slide. Representative images from different cell densities are shown in Fig. S1.

Images of 4 different fields were acquired for each membrane with an optical microscope using a 20× magnification. Each one of the 3 independent OVCAR4 experiments was repeated in triplicates while, for A2780 cells, 2 independent experiments were conducted, each one consisting of 2 replicates.

### Traditional data analysis

The multi-point tool of ImageJ (Waine Rasband, National Institute of Health, USA) was used to manually count the cells. Out of focus regions and cells overlapping the left and bottom edges of the images were excluded from the analysis. The time required to count the cells in each image was manually recorded using a stopwatch.

### Data analysis with I-AbACUS

I-AbACUS is a custom-made program developed in Matlab R2016a (The Mathworks, Inc., Natick, Massachusetts, USA), that was compiled and is freely available at www.marilisacortesi.com as a standalone application or as source code.

The program’s GUI guided the analysis of the data here presented, that were obtained with the default segmentation parameters (15, 9, 11, 17, 19). The scale factor, on the other hand, was varied depending on the technical specifications of the computer.

The counting time was measured with a stopwatch, and includes both the time required by the program to analyze the images and that used to adjust the cell count.

### Statistical methods

The statistical significance of the difference between two variables was evaluated with the Welch’s t-test^13^, that considers the two samples to be independent and does not assume the equality of their variances.

### Image elaboration and segmentation

The strategy adopted to segment the images is the marker controlled watershed transform^14^ applied to the gradient of the saturation channel of the image. Prior to segmentation, the background is uniformed, by masking the current image with its coarse segmentation obtained applying the Otsu’s method^15^ and markers for the foreground and background regions are identified. This procedure suppresses spurious local minima of the gradient and improves the segmentation. In the present study the foreground markers were obtained using the morphological operators opening by reconstruction and closing by reconstruction combined with other grayscale morphological operations aimed to remove the smallest markers. Segmenting the reconstructed image with the Otsu’s method^15^ allows the determination of the regions containing the background markers. These are then obtained by computing the skeleton by influence zone, that is identifying the ridge lines of the watershed transform computed on the distance transform of the segmented image. Finally, the gradient of the saturation is modified, so that the identified markers are the only minimal, and the watershed transform is computed, resulting in the segmented image.

Each foreground region is then analyzed to determine whether or not it is a cell. In I-AbACUS two alternative functions execute this step, an EF or a trained SVM. They both consider, for each region, three characteristics; its area, the interquartile range (IQR) of the values of its pixels and its circularity (*C* = 4πA/P), where A and P represent the area and the perimeter of the object. When the EF is used, an object is considered to be a cell if at least two of the previously mentioned characteristics have values within predefined ranges. Specifically, the area must be between 10^2^ and 10^5^ pixels, the difference between the circularity of the object and that of a circle must be below 1 and the IQR must be above 20 and below 50. Alternatively, the user can employ a trained SVM, a classifier that has been optimized to distinguish a specific kind of cells on the basis of the three aforementioned characteristics. To make this option available even when the two classes are non-linearly separable, the kernel method is applied, using a polynomial kernel^16,17^.

In I-AbACUS the user can adjust the cell count by selecting, for each incorrectly segmented and/or classified region, one of the 4 proposed alternatives. These are obtained by changing the segmentation parameter that, being related to the target dimension of the foreground regions, leads to either a more refined segmentation, that captures the smaller cells or to a coarser one, that allows the identification of larger cells. To minimize the elaboration time and eliminate any delay in this phase of the analysis, the 5 alternative segmentations are implemented as a parallel process. If none of the alternatives is correct the user can manually provide the number of cells in the selected region.

At the end of the elaboration the resulting cell counts can be saved as an excel spreadsheet that can be used for further analysis. The main steps of the analysis with I-AbACUS are summarized in Fig. S2.

### Cell count analysis

The cell counts were analyzed with custom made software written in Python 2.7.12. Mean and standard deviation were calculated for every condition and the percentage error was obtained considering the manual count as theoretical value.

The linear relations reported in the scatter plots were identified with a least square regression, while the correlation was computed as the Pearson’s coefficient.

### Learning algorithm

One of the two cell classification methods implemented in I-AbACUS is a SVM, a supervised learning model that, provided an adequate training set, can be used to improve the recognition of a specific type of cells^16^.

The trained SVM used in this study was obtained running the I-AbACUS learning algorithm on a training set composed of 160 images (48 from A2780 cells and 112 from OVCAR4 cells), completely independent from the test set used during the I-AbACUS’s validation. These images comprised a significant range of densities and obtained a percentage error of less than 35% in over 90% of the images, when analyzed both with the traditional approach and with I-AbACUS.

### Inter-operator variability

To test whether the use of I-AbACUS could reduce the result’s dependence on the operator, two sets of images of OVCAR4 cells (4 or 5 for each tested condition) were counted by five additional operators (besides the I-AbACUS expert) with different levels of experience with the assay and its traditional analysis. One of them was expert on the task, two had never done it before (“naive operator”) and the others had had some experience in the past (“medium operator”). They were asked to count the cells both manually, using ImageJ, and with I-AbACUS, using the EF, and record both the number of cells in each image and the counting time.

The results are presented as the difference between the average cell count obtained for each condition by the naive and medium experience operators and that of the experienced operator. All results presented are normalized with respect to the results obtained by the I-AbACUS expert operator and the independent two-sample t-test was used to evaluate the statistical difference between the results obtained with the two approaches.

### Intra-operator variability

One operator for each level of experience was asked to count again 9 images from the dataset scored during the Inter-Operator variability test.

For this test only three cellular densities were considered (1 × 10^5^, 5 × 10^5^ and 9 × 10^5^ cells/mL) and 3 images for each condition were selected.

As described for the previous test, all the images were also counted manually, and the counting times were recorded with a stopwatch.

The results are presented graphically as a scatter plot, in which the percentage errors between the first and second count of the same image are reported and also summarized as the median percentage error for each counting technique.

## Electronic supplementary material

Supplementary Information
