# Supplementary material for: I-AbACUS: a Reliable Software Tool for the Semi-Automatic Analysis of Invasion and Migration Transwell Assays
Source: Sci Rep. 2018 Feb 28;8:3814. doi: 10.1038/s41598-018-22091-5 (PMC5830488; doi:10.1038/s41598-018-22091-5)
Supplement: Supplementary file 1 — Supplementary Information [file 41598_2018_22091_MOESM1_ESM.pdf]

Supplementary Material: I-AbACUS: a Reliable Software Tool for the Semi-Automatic Analysis of Invasion and Migration Transwell Assays

Marilisa Cortesi[1,*], Estelle Llamosas[2], Claire E. Henry[2], Raani-Yogeeta A. Kumaran[2], Benedict Ng[3], Janet Youkhana[3], and Caroline E. Ford[2,*]

[1]Laboratory of Cellular and Molecular Engineering "S. Cavalcanti", Department of Electrical, Electronic and Information Engineering "G. Marconi" (DEI), University of Bologna, Cesena, Italy.

[2]Gynaecological Cancer Research Group, Lowy Cancer Research Centre and School of Women's and Children's Health, Faculty of Medicine, University of New South Wales, Australia.

[3]Adult Cancer Program, Lowy Cancer Research Center, Prince of Wales Clinical School, University of New South Wales, Australia. *marilisa.cortesi2@unibo.it, caroline.ford@unsw.edu.au

Supplementary Methods

Description of I-AbACUS

I-AbACUS was developed to integrate within a single framework all the phases of the analysis of a transwell migration/invasion assays (Fig. S2). Upon starting the program, the user is asked to provide the details of the experiment (number of conditions and replicates, location of the images), through an interactive format that adapts to every experiment design. The only requirement in this phase is that the images must be organized according to the condition and replicate they refer to. Specifically, the user must create a folder for every tested condition, each containing one directory for every replicate that, in turn, comprise the corresponding images. To ensure the correct association between the outlined experiment structure and the corresponding images, and thus the cell counts, the replicate folders must have a name that ends with "_replicateNumber" where replicateNumber is an integer representing the index of the corresponding replicate.

Once the experiment structure has been outlined the analysis proceeds with the segmentation of the images and the classification of the foreground objects. The former is realized applying the marker controlled watershed transform to the saturation channel of the images coded in the HSV colour space. This algorithm [1], is particularly effective with images in which the foreground objects are contiguous, like in the present case as it considers the local minima of the gradient of the image to be metaphorical water sources and it draws the edges where water from two adjacent basins meets. As described in the main text, the segmentation is preceded by the marking of each foreground and background region, using morphological operators. This operation improves the result by suppressing the spurious minima without affecting the cells shape.

After the segmentation has been completed, every foreground object is analyzed to determine if it is a cell. In I-AbACUS there are two alternative functions that realize this task, an EF and a

classifier based on a trained SVM. The former was designed to be generally applicable to different types of cells, while the latter can be used to improve the classification of a specific type of cells. Both methods consider 3 characteristics for each foreground region, its area, that is used to exclude debris and small irregularities, the difference between the object's circularity and that of a circle, that prevents pores from being recognized and the interquartile range of the values of its pixels. This is a measure of the dispersion of the pixel values within the object and can be used to exclude debris and out of focus cells.

The trained SVM can be obtained through the learning algorithm integrated within I-AbACUS. SVMs are supervised learning models, thus they require a training set to develop the classifying criterion. This can be developed by analyzing images with the EF of I-AbACUS, since a text file, containing the morphological characteristics of the segmented objects (area, circularity, saturation's IQR) and their classification (1 for cells and 0 for non-cells) will be automatically saved in a folder named "Results" in the same path as the I-AbACUS code. The SVM can then be trained, through the "Learning Algorithm" tab of the I-AbACUS GUI, simply selecting the folder containing the appropriate files.

The classification of the foreground objects leads to the suggested cell count that the user can revise by selecting an area of the image that is not correctly segmented and or classified. This causes another window to open, in which four alternative segmentations for that specific region are proposed. These are obtained with different parameters that, being related to the size of the target objects, result in either a finer or coarser segmentation. If none of the alternatives is correct, the user can manually input the number of cells in the selected area. The cell count is automatically updated after each modification and retained within I-AbACUS for the duration of the analysis. After all the images have been segmented the user is given the option to visualize the results, in tabular form and as a histogram, in which the average and standard deviation of the cell count for each replicate and condition is reported, and then save the results as an excel file.

The training of the SVM is a separate step from the image analysis workflow and can be run through the dedicated panel in the I-AbACUS GUI. It executes a procedure that, using the provided examples, builds a model able to assign new objects to the appropriate category. SVMs are linear classifiers that identify the hyperplane that classifies the provided data with the largest margin, but they can also be applied to non-linearly separable examples through the kernel method. It consists in using a polynomial kernel to map the original feature space to one of higher dimension, where the data are linearly separable, and then computing the maximum margin hyperplane. Once the model has been identified I-AbACUS saves it in .mat file, requesting the user to select the desired name and location.

References

[1] Meyer, F. & Beucher, S. Morphological segmentation. Journal of Visual Communication and Image Representation 18 (2011).

Supplementary Figure Legends

Figure S1. Images representative of the ones used in this study. For the validation of I-AbACUS two ovarian cancer cell lines (OVCAR4, A2780) were used. The cells that had

crossed the membrane were fixed, stained with crystal violet and images of four different fields were acquired with an optical microscope. Since OVCAR4 cells are larger and more diverse than A2780, they have been tested more extensively with I-AbACUS. In particular multiple initial cell densities, three of which are here reported ($1x10^5$, $5x10^5$, $9x10^5$ cells/mL) have been used. A2780 cells, on the other hand, were only tested at the optimal cell density of $5x10^5$cells/mL.

Figure S2. Flowchart detailing the main steps of the analysis with I-AbACUS. After checking the program's settings the user is asked to detail the experiment's setup and provide the location of the images to analyze. Pressing the "Next Image" button in the Single Image Analysis tab starts the segmentation of the first image. Once the image has been analysed the user can change the cell count by selecting an incorrectly segmented/classified region and choosing one of the four alternatives. Alternatively, it is possible to directly input the number of cells in the region through the specific input field. These steps are repeated for every incorrectly recognized area and for every image. At the end of the elaboration the user can visualize the results and save them on the hard drive.

Supplementary Figure 1

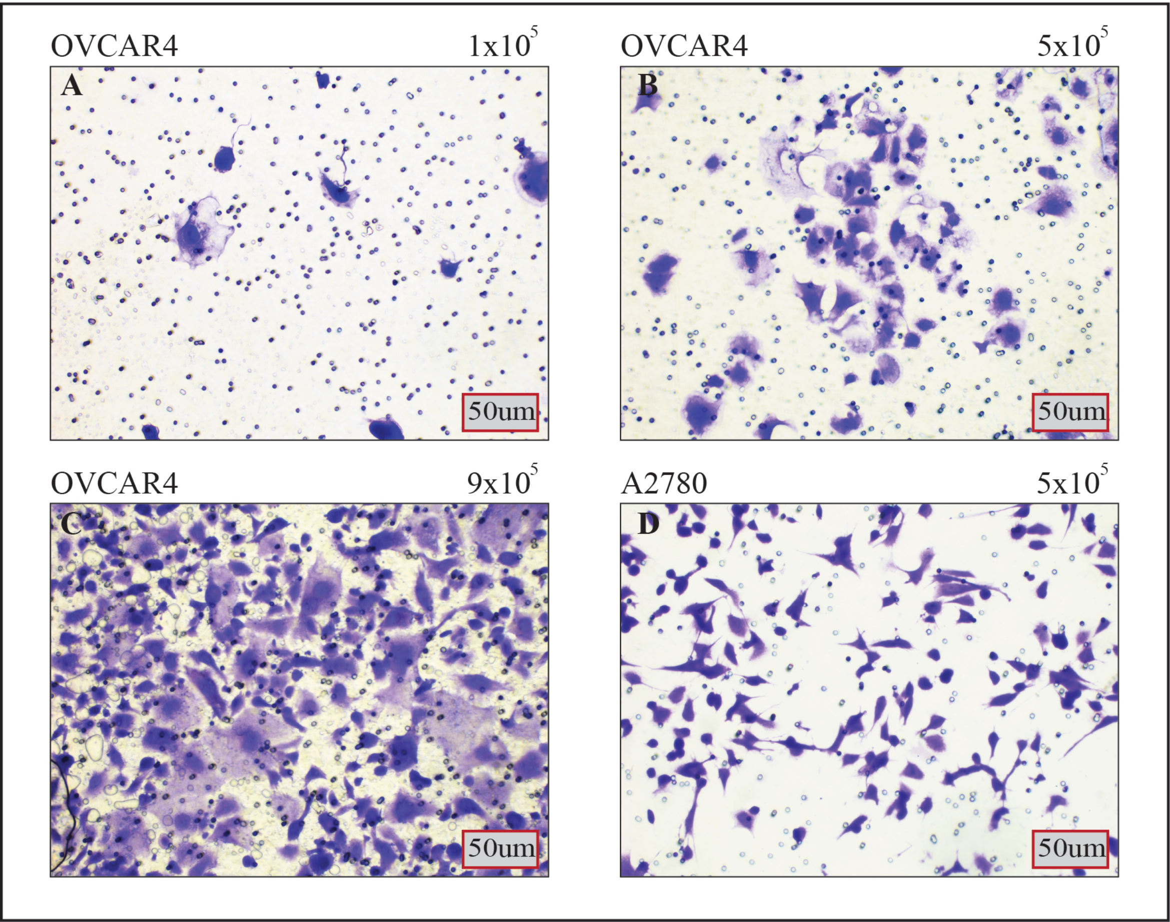

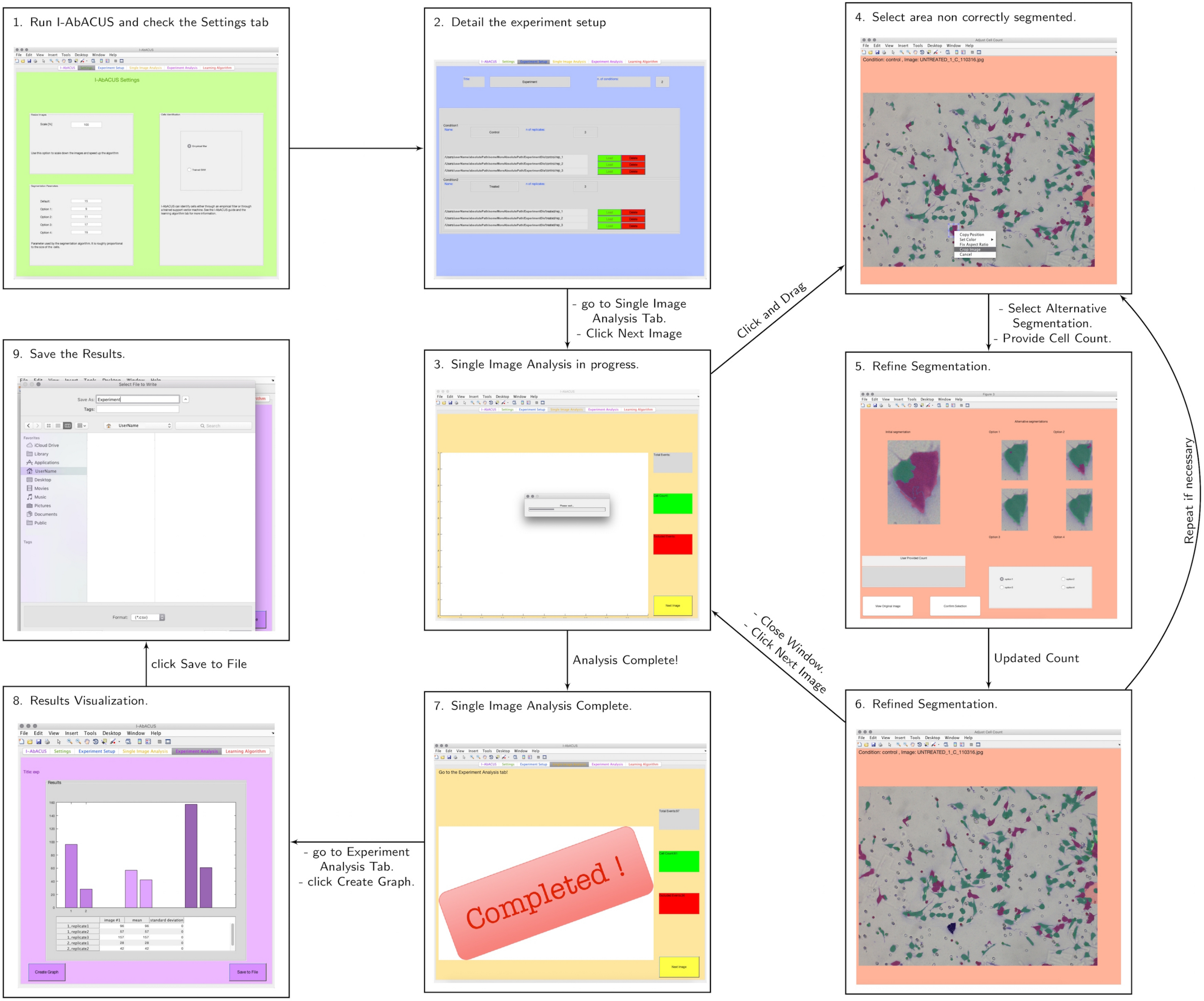
1. Run I-AbACUS and check the Settings tab
2. Detail the experiment setup
- go to Single Image Analysis Tab.
- Click Next Image
3. Single Image Analysis in progress.
Click and Drag
4. Select area non correctly segmented.
- Select Alternative Segmentation.
- Provide Cell Count.
5. Refine Segmentation.
Updated Count
6. Refined Segmentation.
Repeat if necessary
- Close Window.
- Click Next Image
Analysis Complete!
7. Single Image Analysis Complete.
Completed !
- go to Experiment Analysis Tab.
- click Create Graph.
8. Results Visualization.
click Save to File
9. Save the Results.
